# MiR-133b-3p attenuates angiotensin II-induced cardiac hypertrophy through the inhibition of apoptosis by targeting *CDIP1*: MiR-133b-3p attenuates angiotensin II-induced cardiac hypertrophy

**DOI:** 10.3724/abbs.2024181

**Published:** 2025-02-12

**Authors:** Jiwei Gu, Zhen Li, Xinyi Li, Ziyao Yang, Xi Xu, Yanjia Wang, Xiaohan Li, Kaiyue Qin, Guizhong Li, Li Xue, Xiaoling Yang

**Affiliations:** 1 NHC Key Laboratory of Metabolic Cardiovascular Diseases Research Ningxia Medical University Yinchuan 750004 China; 2 Department of Cardiovascular Surgery General Hospital of Ningxia Medical University Yinchuan 750004 China; 3 Department of Second Clinical Medicine Ningxia Medical University Yinchuan 750004 China; 4 School of Basic Medical Sciences Ningxia Medical University Yinchuan 750004 China; 5 Department of Cardiology General Hospital of Ningxia Medical University Yinchuan 750004 China; 6 Ningxia Key Laboratory of Vascular Injury and Repair Research Ningxia Medical University Yinchuan 750004 China

**Keywords:** cardiac hypertrophy, apoptosis, miR-133b-3p, *CDIP1*

## Abstract

MicroRNAs (miRNAs) have emerged as essential regulators that play important roles in the development of multiple systems. Recent studies have identified significant roles for miRNAs in the progression of cardiac hypertrophy. This study aims to investigate the effects of miR-133b-3p on angiotensin II (Ang II)-induced cardiac hypertrophy and apoptosis, as well as explore its underlying mechanisms. Our experimental results reveal that miR-133b-3p expression is significantly decreased in both animal and cell models of cardiac hypertrophy induced by Ang II. Overexpression of miR-133b-3p reverses the hypertrophic manifestations and apoptosis induced by Ang II. Through bioinformatics analysis and dual-luciferase reporter assays, *CDIP1* (cell death inducing p53 target 1) is identified as a direct target of miR-133b-3p, and the overexpression of miR-133b-3p reduces *CDIP1* expression. Additionally, *CDIP1* silencing suppresses cardiomyocyte hypertrophy and apoptosis induced by Ang II. In summary, these results suggest that miR-133b-3p may serve as a potential diagnostic marker for cardiac hypertrophy and that the upregulation of miR-133b-3p inhibits cardiac hypertrophy by targeting *CDIP1*.

## Introduction

Pathological cardiac hypertrophy is an adaptive response of the myocardium to pressure overload in the heart and is commonly observed in patients with hypertension, myocardial infarction, and valvular diseases [ 1, 2] . Pathological cardiac hypertrophy often precedes overt heart failure and is an independent prognosticator of cardiovascular mortality [3]. The development of left ventricle hypertrophy is predominantly due to increased cardiomyocyte size. It is characterized by an overall increase in protein synthesis and fetal gene expression, including that of atrial natriuretic peptide (ANP) and brain natriuretic peptide (BNP) [ 4, 5] . The renin-angiotensin system (RAS) is a key player in controlling homeostasis in the cardiovascular system, and angiotensin (Ang) II is the representative hormone in the RAS. As a vasopressor, Ang II contributes to pathological cardiac hypertrophy and the resulting heart failure indirectly via increased blood pressure and/or direct action on cardiomyocytes [6]. Recent reports have revealed that apoptosis is involved in regulating the progression of multiple cardiovascular diseases, including cardiac hypertrophy. However, the underlying mechanisms remain poorly understood [7].

MicroRNAs (miRNAs) are a group of endogenous noncoding single-stranded small-molecule RNAs comprising 21–23 nucleotides that play a master role in regulating gene expression [8]. Increasing evidence shows that miRNAs are involved in the posttranscriptional regulation of gene expression to maintain cardiac homeostasis [9]. Recent studies have indicated that changes in the expression levels of miRNAs may positively or negatively regulate cardiac hypertrophy and may be promising therapeutic targets for intervention. For example, miR-30d was decreased in both murine and neonatal rat cardiomyocyte models of hypertrophy, and overexpression of miR-30d ameliorated phenylephrine- and Ang II-induced cardiac hypertrophy [10]. Researchers have discovered that cholesterol-containing nanocarriers can efficiently deliver inhibitors of miR-182 into the heart to significantly suppress cardiac hypertrophy [11]. Additionally, systemic knockout of miR-27b attenuates Ang II-mediated pathological cardiac hypertrophy and myocardial fibrosis by targeting the *FGF1* gene [12]. MiR-133b is frequently abnormally expressed in various kinds of human cancer, and its complex regulatory networks affect the tumorigenicity and development of malignant tumors [ 13– 16] . Yu *et al*. [16] reported that the level of miR-133b-3p was significantly decreased during the postnatal heart growth period. In addition, it has also been reported that the overexpression of miR-133b-3p promoted the apoptosis of high glucose-treated mouse retinal microvascular endothelial cells [17]. However, whether miR-133b-3p could be a therapeutic target for pathological cardiac hypertrophy is undetermined.

Cell death-inducing p53 target 1 (CDIP1) is a protein that plays a role in apoptosis or programmed cell death, particularly in response to cellular stress and DNA damage [18]. CDIP1 induces apoptosis primarily through interactions with Bcl-2 family proteins, particularly proapoptotic proteins such as Bax and Bak. These interactions lead to mitochondrial outer membrane permeabilization (MOMP), cytochrome c release, and subsequent activation of caspases, which execute the apoptotic program [19]. It has been reported that dysregulation or loss of CDIP1 function can contribute to cancer progression. Reduced expression or activity of CDIP1 may impair the ability of cells to undergo apoptosis in response to stress, allowing damaged cells to survive and potentially accumulate additional mutations that promote malignancy [ 20, 21] . However, whether CDIP1 is involved in Ang II-induced cardiomyocyte apoptosis remains to be explored.

In this study, we aimed to investigate the expression pattern and functional role of miR-133b-3p in Ang II-induced cardiac hypertrophy and the possible contribution of its predicted target *CDIP1* to cardiac hypertrophy progression.

## Materials and Methods

### Animal model

A total of 16 six-week-old male C57BL/6 mice, each weighing 20 ± 2 g, were purchased from Ningxia Medical University Laboratory Animal Centre (Yinchuan, China). After 2 weeks of adaptation, the mice were randomly assigned to the Ang II infusion group ( *n*  = 8) or the sham group ( *n*  = 8). Myocardial hypertrophy in mice was induced by chronic infusion of Ang II (Ang II dissolved in PBS with 10 μM acetic acid) at a dose of 500 ng/kg/min using a subcutaneously implanted minipump for 30 days. The mice that were infused with the same dose of PBS served as the sham group. After the mice were anaesthetized with 3.0% isoflurane, the hearts were isolated and weighed on an analytical balance, and the heart-to-body weight (HW/BW) ratio was calculated. The animal experiment was approved by the Ningxia Medical University Animal Care and Use Committee (ethics approval number: 2020--556), and the guidelines of the Ningxia Medical University Animal Care Committee were strictly followed.

### Echocardiography

Cardiac function in the mice was assessed using the Vevo 770 imaging system (Visual Sonics, Toronto, Canada), which is equipped with a 40 MHz transducer. The mice were initially anaesthetized with 2% isoflurane, with anaesthesia subsequently maintained at a level of 0.5% isoflurane. The mice were then positioned in a supine posture on a dedicated monitoring table. We employed B-mode echocardiography to capture the relevant cardiac images. These images were then analyzed with Vevo Lab software, through which we quantified various cardiac functional indices, including the left ventricular ejection fraction (EF%), left ventricular fractional shortening (LVFS%), dimensions of the left ventricular posterior wall in diastole (LVPWd), and systole (LVPWs). All the data from all the mice were measured at least 3 times, and the data were statistically analyzed.

### Cell culture and treatment

Rat cardiomyocytes (H9c2 cells) purchased from the Cell Bank of the Chinese Academy of Sciences (Shanghai, China) were cultured in DMEM supplemented with 10% fetal bovine serum (FBS) and 100 IU/mL penicillin-streptomycin at 37°C in 5% CO _2_. H9c2 cells were subjected to 48 h of treatment with 1.0 μML Ang II or PBS. *CDIP1* siRNA and siRNA negative controls were purchased from GenePharma (Shanghai, China). The miR-133b-3p mimic, miR-133b-3p inhibitor, and miRNA negative control (miR-NC) were purchased from RiboBio (Guangzhou, China). Cell transfection was performed with 20 μM *CDIP1*-siRNA, 20 μM miR-133b-3p mimic or inhibitor using Lipofectamine 2000 (Invitrogen, Carlsbad, USA) according to the manufacturer’s instructions. The experiments were performed 48 h posttransfection. The siRNA sequences used are listed in Table 1.

**Table:** **Table 1** Sequences of miR133b-3p NC, miR-133b-3p mimic, miR-133b-3p inhibitor, si-NC, and si-CDIP1-1-3

Name	Species	Sequence (5′→3′)
miR133b-3p NC	Rat	Sense: UUCUCCCGUGUCACGUTT
	Antisense: ACGUGACACGUUCGGAGAATT
miR-133b-3p mimic	Rat	Sense: UUUGGUCCCCUUCAACCAGCUA
	Antisense: GCUGGUUGAAGGGACCAAAUU
miR-133b-3p inhibitor	Rat	Sense: UAGCUGGUUGAAGGGGACCAAA
si-NC	Rat	Sense: UUCUCCCGUGUCACGUTT
		Antisense: ACGUGACACGUUCGGAGAATT
si-CDIP1-1	Rat	Sense: ACCCACCUAUGGGCUAUUATT
		Antisense: UAAUAGCCCAUAGGUGGGUTT
si-CDIP1-2	Rat	Sense: UACUGCAGGGAGAGAUCUUTT
		Antisense: AAGAUCUCUCCCUGCAGUATT
si-CDIP1-3	Rat	Sense: GCCUACAUCUGCACAUACATT
		Antisense: UGUAUGUGCAGAUGUAGGCTT

### Hematoxylin and eosin (HE) and Wheat germ agglutinin (WGA) staining

Excised mouse hearts were briefly rinsed with cold PBS and immediately fixed in 4% paraformaldehyde at room temperature for 24 h. The fixed tissues were then dehydrated and embedded in paraffin wax. The tissues were subsequently sectioned into 4 μm thick slices and dried at 60˚C for 30 min. Deparaffinization was performed using xylene, followed by rehydration through a series of graded alcohols (100%, 90%, 80%, and 70%). For HE staining, the sections were stained with hematoxylin (Solarbio, Beijing, China) for 10 min. Differentiation was done in 1% hydrochloric acid in 70% ethanol for 1 min, followed by another rinse. Counterstaining was performed using eosin (Solarbio) for 2 min. Rapid dehydration was carried out using 95% and 100% alcohol. Finally, the slides were observed and photographed under a light microscope (Olympus, Tokyo, Japan). For WGA staining, the sections were stained with WGA working solution (Vectorlabs, Tokyo, Japan) and incubated at 37°C for 30 min. After being sealed with neutral gum, the slides were observed and photographed under a light microscope (Olympus).

### Masson staining

Masson staining was used to detect fibrosis in the cardiac tissue according to the manufacturer′s instructions of the Masson′s trichrome staining kit (Solarbio). In brief, the sections were stained with hematoxylin for 5 min, ponceau acid fuchsin for 5 min, and fast green FCF for 1 min, after which the slides were observed and photographed under a light microscope (Olympus).

### Immunofluorescence

Cardiomyocytes were fixed in 4% paraformaldehyde for 20 min and permeabilized with 0.2% Triton X-100 for 20 min. After being blocked with 10% goat serum, the cells were incubated with anti-cardiac troponin T antibodies (cTnT; Proteintech, Wuhan, China) at 4°C overnight. Next, the cells were washed three times with PBS and then incubated with fluorescein-conjugated secondary antibodies (ZEN-Biologics, Chengdu, China) for 2 h at room temperature. The cell nuclei were stained by incubation with 4,6-diamidino-2-phenylindole (DAPI; Bioss, Woburn, USA). Fluorescence was detected and photographed by confocal microscopy (Zeiss, Jena, Germany). The fluorescence intensity was quantified using ImageJ software.

### Transmission electron microscopy (TEM)

The ventricle samples were fixed in 2% glutaraldehyde, and sodium cacodylate buffer was used to wash the ventricle samples 3 times for 10 min each. The ventricle samples were fixed with 1% osmium tetroxide for 1 h. Then, the ventricle samples were dehydrated with an increasing concentration gradient of ethanol and propylene oxide. The ventricle samples were then embedded, cut into 50 nm sections, and stained with uranyl acetate solution and lead citrate solution. The pathological changes were examined by a JEOL transmission electron microscope (Tokyo, Japan).

### Western blot analysis

Tissues and cells were lysed in lysis buffer (KeyGEN Biotech, Nanjing, China) on ice. Lysed cells were centrifuged at 16048 g for 15 min, and the protein concentration in the supernatant was quantified by a BCA protein assay kit (KeyGEN Biotech, Nanjing, China). A total of 30 μg of protein was separated via 10% sodium dodecyl sulfate-polyacrylamide gel electrophoresis (SDS-PAGE) and then transferred to polyvinylidene fluoride (PVDF) membranes (Millipore, Billerica, USA). After being blocked with 5% non-fat milk for 1 h, the membrane was then incubated with the following primary antibodies: anti-CDIP1 (ABclonal, Wuhan, China; rabbit, 1:500), anti-ANP (ABclonal; rabbit, 1:1000), anti-BNP (Affinity, Cincinnati, USA; rabbit, 1:1000), anti-Bax (Affinity; rabbit, 1:1000), anti-cleaved caspase-3 (Wanleibio Shenyang, China; rabbit, 1:1000), anti-Bcl-2 (Affinity; rabbit, 1:1000), and anti-β-actin (Abways, Shanghai, China;rabbit, 1:10000) overnight at 4°C. After 3 washes with TBST, the membranes were incubated with goat anti-rabbit IgG H&L (ABclonal; 1:2500) or goat anti-mouse IgG H&L (ABclonal; 1:2500) at room temperature for 1 h. Protein expression was then detected using the chemiluminescence kit (KeyGEN). The optical density of each band was quantified using densitometry and normalized to that of a β-actin loading control.

### Immunohistochemistry (IHC)

The left ventricular sections were deparaffinized in xylene and rehydrated in an ethanol gradient. Then, the sections were blocked for 30 min in 10% normal goat serum and were separately incubated with primary antibodies directed against Bax, cleaved caspase-3, and Bcl-2 described above at 37°C for 3 h. After washing, the sections were incubated with secondary antibodies and diaminobenzidine (DAB) at 37°C. The slides were washed and counterstained with hematoxylin, and images were captured under a light microscope (Olympus).

### Quantitative real-time polymerase chain reaction (qRT-PCR) analysis

Total RNA was extracted from tissues or cells using the RNA isolation kit (TIANGEN, Beijing, China) according to the manufacturer’s protocol. After the quantification of RNA, cDNA was synthesized using the reverse transcription kit (Takara, Tokyo, Japan). The cDNA was then amplified by PCR in a real-time cycler using TBGreen Fast qPCR mix (Takara). All the experiments were performed in triplicate, and the data were normalized to *U6* or *GAPDH*. The following primers were used for PCR: *miR-133b-3p* forward primer: 5′-TGAGCGCTTTTGGTCCCCTTC-3′ and reverse primer: 5′-ATCCAGTGCAGGGTCCGAGG-3′; *U6* forward primer: 5′-ATATATGGACGCTTCAATT-3′ and reverse primer: 5′-AACGCTTCGAATGCTTGT-3′; *CDIP1* forward primer: 5′-CCTGTGTGTTGCTTGGACATTTGC-3′ and reverse primer: 5′-GGAAGAATAAGGACCAGCCTGAACC-3′; and *GAPD*H forward primer: 5′-GACATGCCGCCTGGAGAAAC-3′ and reverse primer: 5′-AGCCCAGGATGCCCTTTAGT-3′.

### Dual-luciferase assay

The target genes of miR-133b-3p were predicted by the online web tool TargetScan ( http://www.targetscan.org). A dual-luciferase assay was used to validate the predicted connection between miR-133b-3p and *CDIP1*. Briefly, the sequence containing the wild-type binding site in the 3ʹ-untranslated region (3′UTR) of *CDIP1* or the sequence with a mutated binding site was cloned and inserted into the PmirGLO firefly luciferase vector (Promega, Madison, USA). The *CDIP1*-WT and *CDIP1*-MUT plasmids with either the miR-133b-3p mimic or the miR-NC were delivered into H9c2 and HEK293T cells [Fudan IBS Cell Center (FDCC; Shanghai, China)] by Lipofectamine 2000 (Invitrogen) for 48 h. Subsequently, the H9c2 and HEK293T cell lysates were added to Renilla luciferase or firefly luciferase, and the relative luciferase activities were measured using Dual-Luciferase Reporter Assay kit (Promega) on a microplate reader (BioTek, Winooski, USA).

### Statistical analysis

All the results were analyzed with GraphPad Prism 7.0 software. The data in this study are presented as the mean ± standard deviation (SD) from at least three independent experiments. Single parameters were compared between two groups via paired Student’s *t* test. One‐way analysis of variance (ANOVA) was used to compare the means of multiple groups, followed by Newman-Keuls test. A value of *P*  < 0.05 was considered statistically significant.

## Results

### Ang II-induced cardiac hypertrophy and apoptosis *in vivo* and *in vitro*

**Figure 1:**
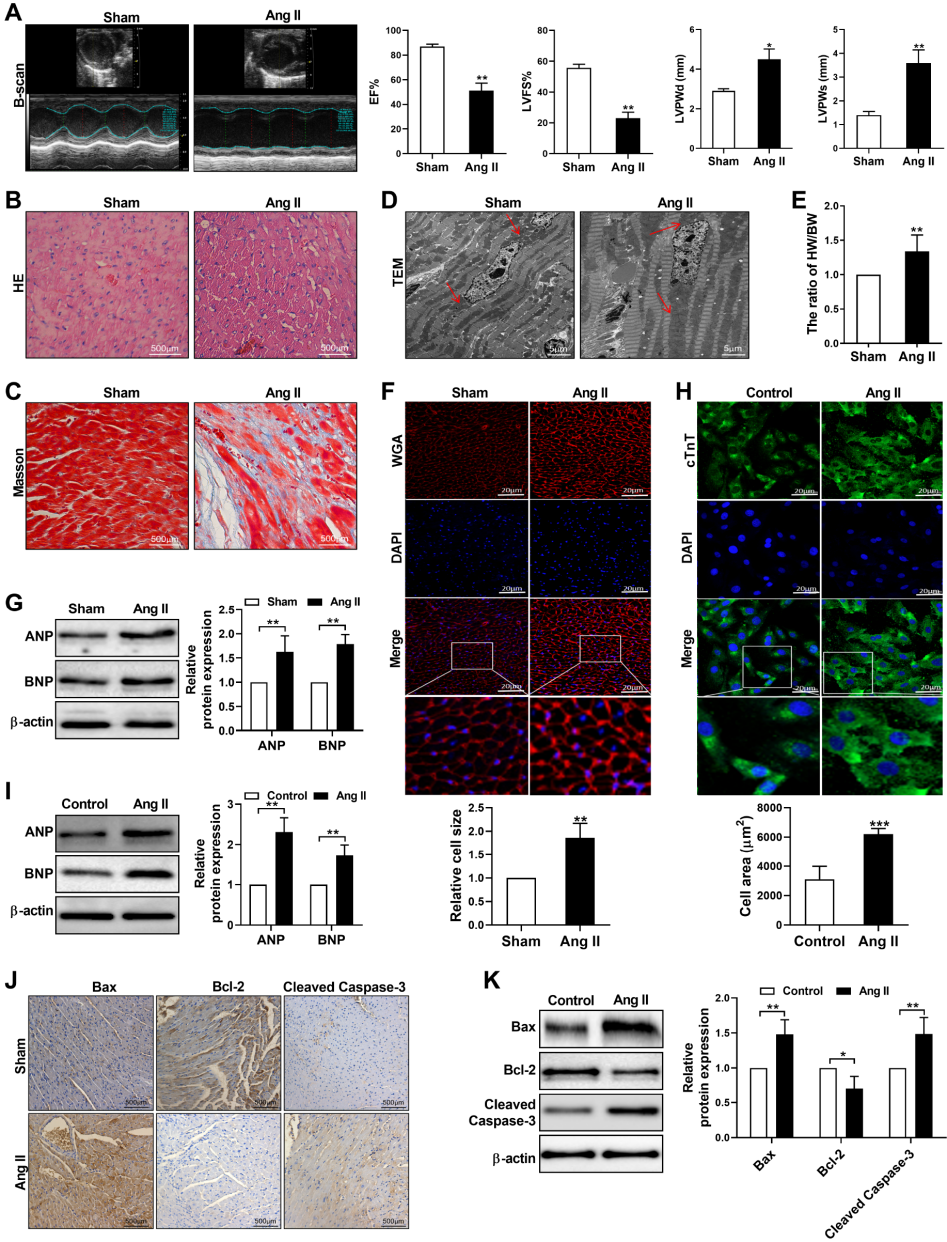
Figure 1 Ang II induced cardiac hypertrophy and apoptosis (A) Representative B-mode images and cardiac function indicators (EF%, LVFS%, LVPWd, and LVPWs) measured by echocardiography in sham and Ang II-infused mice. (B,C) H&E and Masson staining of the left ventricle in sham and Ang II-infused mice. Scale bar: 500 μm. (D) Representative images of the left ventricle of sham and Ang II-infused mice were captured via transmission electron microscopy. Scale bar: 5 μm. (E) Heart-to-body weight (HW/BW) ratios of sham and Ang II-infused mice. (F) Representative images of WGA staining (red) of the left ventricle in sham and Ang II-infused mice. Nuclei were stained with DAPI (blue). Scale bar: 20 μm. (G) Western blot analysis of ANP and BNP expressions in ventricle samples from sham and Ang II-infused mice. (H) Representative immunofluorescence staining of cTnT (green) in cardiomyocytes treated with Ang II. Nuclei were stained with DAPI (blue). Scale bar: 20 μm. (I) Western blot analysis of ANP and BNP expressions in cardiomyocytes treated with Ang II. (J) Immunohistochemical staining of Bax, Bcl-2 and cleaved caspase-3 in sham and Ang II-infused mice. Scale bar: 500 μm. (K) Western blot analysis of Bax, Bcl-2 and cleaved caspase-3 expressions in cardiomyocytes treated with Ang II. The data are presented as the mean ± SD. *P < 0.05, **P < 0.01, ***P < 0.001.

To ensure the accuracy of the experiment, we evaluated whether the cardiac hypertrophy models were successfully established both *in vivo* and *in vitro*. First, we performed echocardiography to measure the cardiac parameters of the mice after 30 days of Ang II infusion. Compared with those in the sham group, the left ventricular ejection fraction (EF%) and left ventricular fractional shortening (LVFS%) in the Ang II-infusion group were significantly decreased, whereas the left ventricular posterior wall dimensions (LVPWd) and systole (LVPWs) were increased ( Figure 1A). The HE staining results revealed that the sham group had more compactly and regularly arranged cardiomyocytes with intact nuclei, whereas the arrangement of myocardial cells in the Ang II-infusion group was disordered, and the infiltration of pro-inflammatory cells increased. In addition, some myocardial cells were disrupted in the Ang II-infusion group ( Figure 1B). Masson staining revealed that Ang II infusion resulted in an increase in blue collagen fibres in the myocardial interstitium ( Figure 1C). Furthermore, the ultrastructure of the myocardium, as detected by transmission electron microscopy, indicated that Ang II infusion caused swelling and deformation of the nuclei and mitochondria, with the mitochondria appearing paralyzed ( Figure 1D). Cardiac hypertrophy was further confirmed by an elevated HW/BW ( Figure 1E). In addition, WGA (wheat germ agglutinin) staining revealed that Ang II infusion significantly increased the cross-sectional area of cardiomyocytes in the hypertrophic myocardium ( Figure 1F). We also examined the expressions of hypertrophic marker genes, including atrial natriuretic peptide (ANP) and B-type natriuretic peptide (BNP), determined by western blot analysis. The results revealed significant upregulation of ANP and BNP in ventricle samples from Ang II-infused mice ( Figure 1G). Moreover, an *in vitro* hypertrophic model was established by treating cardiomyocytes with Ang II for 48 h. Compared with that in the control group, the quantification of the cell area by immunofluorescence staining of cardiac troponin T (cTnT) revealed that the cell area in cardiomyocytes treated with Ang II was strongly increased ( Figure 1H). Consistently, the expression levels of ANP and BNP were markedly increased in cardiomyocytes treated with Ang II ( Figure 1I). These results indicated that Ang II-induced cardiac hypertrophy models were successfully established both *in vivo* and *in vitro*. Next, we evaluated the expression of the proapoptotic proteins Bax and cleaved caspase-3 and the antiapoptotic protein Bcl-2 in Ang II-infused mice and in cardiomyocytes treated with Ang II. The results indicated that Bax and cleaved caspase-3 were significantly increased in the model group both *in vivo* and *in vitro*, whereas Bcl-2 expression was dramatically decreased ( Figure 1J,K). Together, these data indicated that Ang II induces cardiac hypertrophy through the promotion of apoptosis.

### Downregulation of miR-133b-3p in cardiac hypertrophy

**Figure 2:**
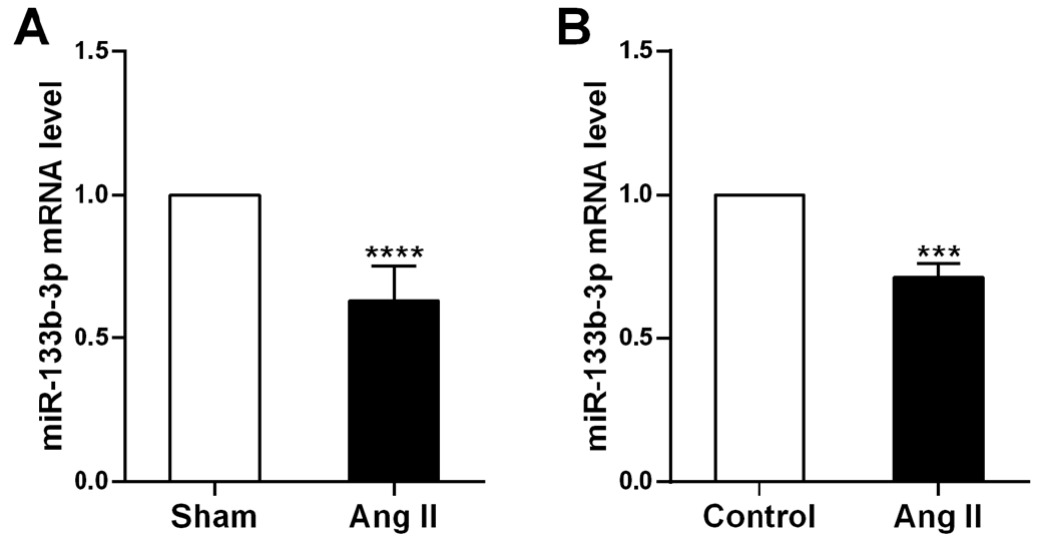
Figure 2 The expression of miR-133b-3p is downregulated in cardiac hypertrophy (A,B) qRT-PCR analysis of miR-133b-3p expression in Ang II-infused mice (A) and cardiomyocytes (B) treated with Ang II. The data are presented as the mean ± SD. ***P < 0.001, ****P < 0.0001.

We next examined the levels of miR-133b-3p in ventricle samples from Ang II-infused mice. Compared with sham treatment, Ang II infusion caused a significant decrease in miR-133b-3p expression ( Figure 2A). Similarly, in a cell model, Ang II treatment significantly reduced miR-133b-3p expression ( Figure 2B). These results suggested that miR-133b-3p may participate in the pathogenesis of cardiac hypertrophy.

### MiR-133b-3p inhibits Ang II-induced cardiomyocyte hypertrophy and apoptosis

**Figure 3:**
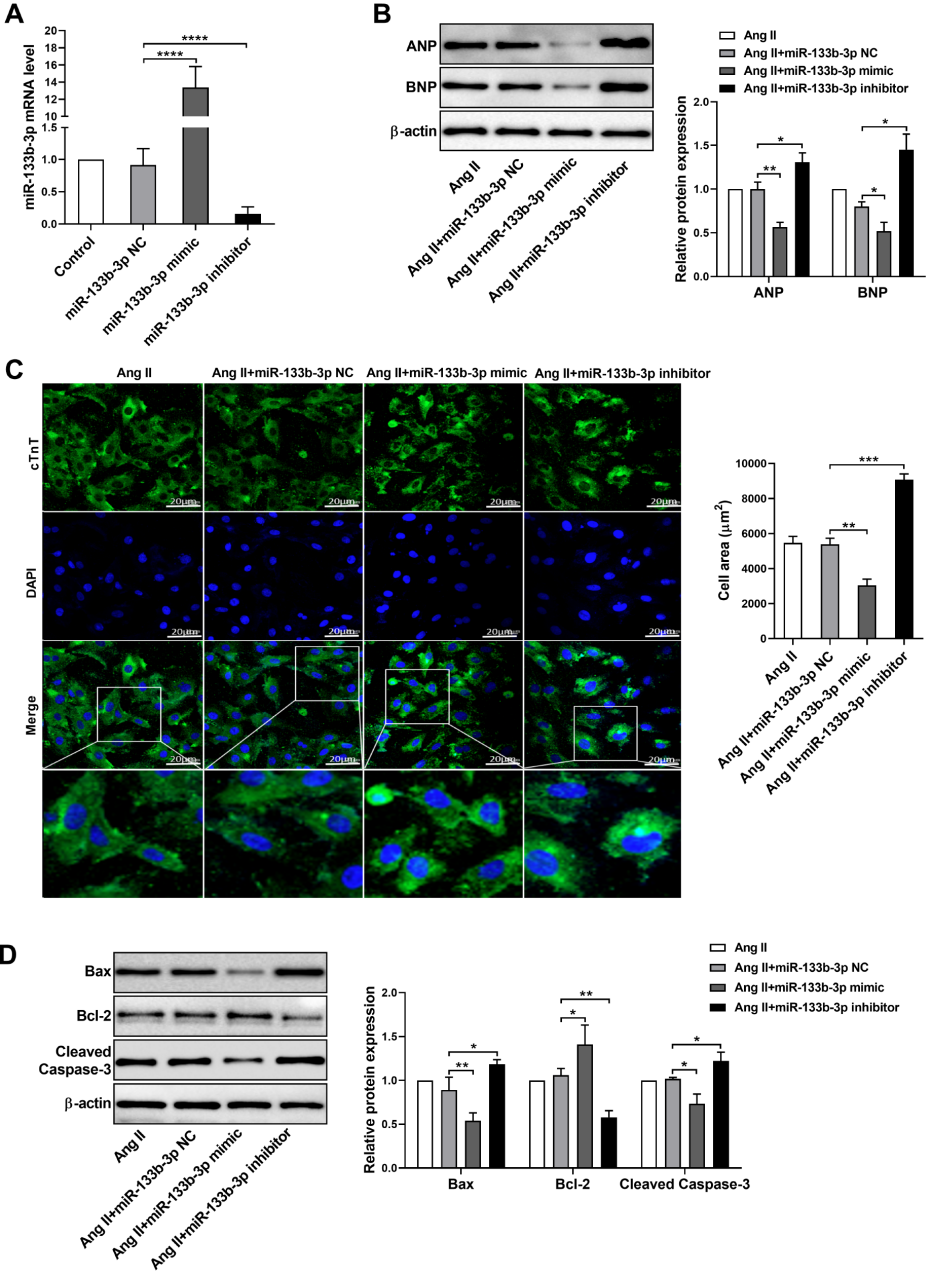
Figure 3 MiR-133b-3p inhibits Ang II-induced cardiomyocyte hypertrophy and apoptosis (A) qRT-PCR analysis of miR-133b-3p expression in cardiomyocytes transfected with the miR-133b-3p mimic, miR-133b-3p inhibitor or negative control (miR-133b-3p NC). (B) Western blot analysis of ANP and BNP expressions in cardiomyocytes transfected with the miR-133b-3p mimic, miR-133b-3p inhibitor or miR-133b-3p NC in the presence of Ang II. (C) Immunofluorescence staining of cTnT (green) in cardiomyocytes transfected with the miR-133b-3p mimic, miR-133b-3p inhibitor or miR-133b-3p NC in the presence of Ang II. Nuclei were stained with DAPI (blue). Scale bar: 20 μm. (D) Western blot analysis of Bax, Bcl-2 and cleaved caspase-3 expressions in cardiomyocytes transfected with the miR-133b-3p mimic, miR-133b-3p inhibitor or miR-133b-3p NC in the presence of Ang II. The data are presented as the mean ± SD. *P < 0.05, **P < 0.01, ***P < 0.001, ****P < 0.0001.

To assess whether miR-133b-3p regulates cardiomyocyte hypertrophy and apoptosis, cardiomyocytes were transfected with either a miR-133b-3p mimic or inhibitor. The qRT-PCR results revealed that cells transfected with the miR-133b-3p mimic presented upregulated miR-133b-3p expression, whereas cells transfected with the miR-133b-3p inhibitor presented reduced miR-133b-3p levels ( Figure 3A). Western blot analysis revealed that the Ang II-induced upregulation of ANP and BNP was suppressed by the overexpression of miR-133b-3p, which was promoted by the miR-133b-3p inhibitor ( Figure 3B). Additionally, immunofluorescence staining for cTnT demonstrated that the overexpression of miR-133b-3p significantly inhibited the increase in cell area induced by Ang II. In contrast, downregulation of miR-133b-3p further enhanced the increase in the cell area induced by Ang II ( Figure 3C). Western blot analysis also revealed that overexpression of miR-133b-3p reduced the expressions of Bax and cleaved caspase-3 and increased Bcl-2 expression, whereas downregulation of miR-133b-3p had the opposite effect ( Figure 3D). Taken together, these findings suggested that the overexpression of miR-133b-3p inhibits Ang II-induced cardiomyocyte hypertrophy by suppressing apoptosis.

### *CDIP1* is a target of miR-133b-3p

**Figure 4:**
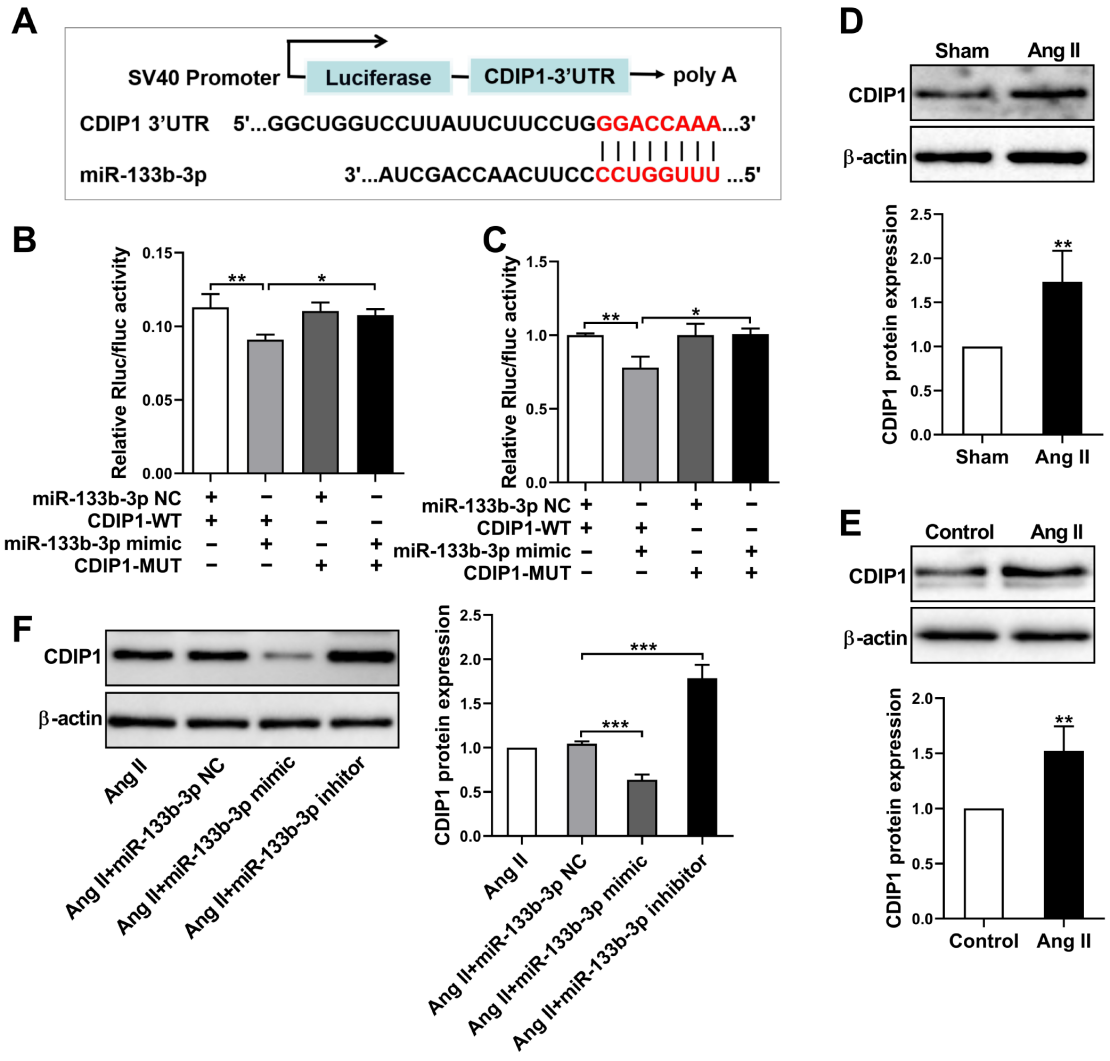
Figure 4 *CDIP1* is a target negatively regulated by miR-133b-3p (A) Bioinformatics analysis of the binding site between the 3′UTR of CDIP1 and miR-133b-3p. (B,C) Dual-luciferase reporter assay using the WT-CDIP1 3′UTR reporter and MUT reporter in the presence of the miR-133b-3p mimic. (D,E) Western blot analysis of CDIP1 expression in Ang II-infused mice and cardiomyocytes treated with Ang II. (F) Western blot analysis of CDIP1 expression in cardiomyocytes transfected with the miR-133b-3p mimic or miR-133b-3p inhibitor in the presence of Ang II. The data are presented as the mean ± SD. *P < 0.05, **P < 0.01, ***P < 0.001.

Since miR-133b-3p plays an important role in cardiac hypertrophy, we used TargetScan to predict the downstream target mRNAs of miR-133b-3p. On the basis of the prediction, we identified a conserved binding site for miR-133b-3p in the 3′UTR of the *CDIP1* gene ( Figure 4A). To further verify that miR-133b-3p directly targets *CDIP1*, we performed luciferase reporter assays in H9c2 cells and HEK293T cells. The results revealed that the activity of the *CDIP1* 3′UTR-WT reporter was significantly inhibited by the miR-133b-3p mimic, whereas mutating the binding sequence had no effect ( Figure 4B,C). These data suggest that miR-133b-3p specifically binds to the 3′UTR of *CDIP1*. We subsequently detected the expression of *CDIP1* in cardiac hypertrophy. Compared with that in control cardiomyocytes, the expression of *CDIP1* in Ang II-infused mice and Ang II-treated cardiomyocytes was significantly greater ( Figure 4D,E). Additionally, transfection with the miR-133b-3p mimic markedly reduced *CDIP1* expression, whereas the miR-133b-3p inhibitor increased *CDIP1* expression, indicating that *CDIP1* is negatively regulated by miR-133b-3p ( Figure 4F). Therefore, *CDIP1* is a target of miR-133b-3p involved in the regulation of cardiac hypertrophy.

### Silencing *CDIP1* suppresses Ang II-induced cardiomyocyte hypertrophy and apoptosis

**Figure 5:**
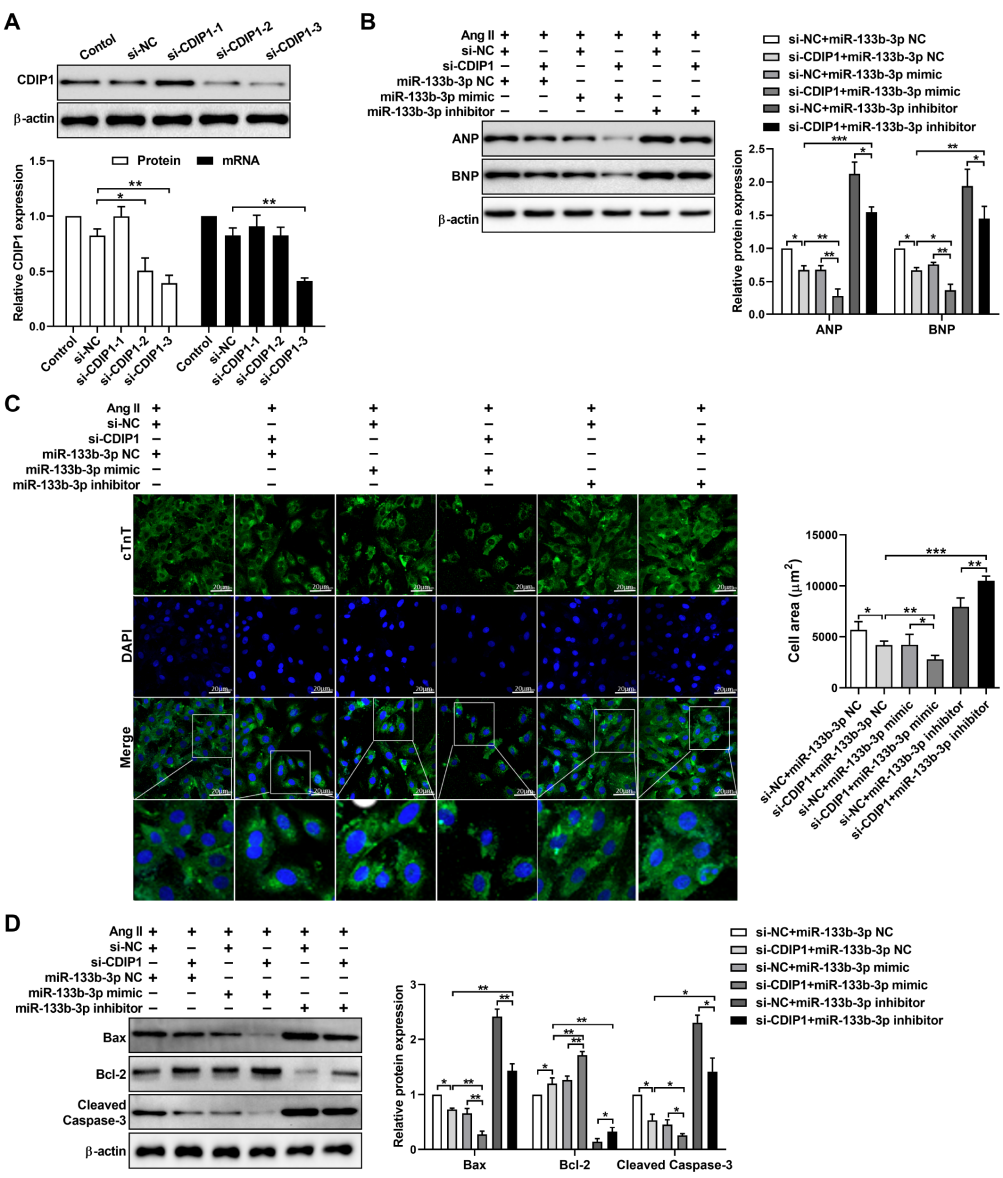
Figure 5 Effects of CDIP1 on cardiomyocyte hypertrophy and apoptosis (A) Western blot and qRT-PCR analysis of CDIP1 expression in cardiomyocytes transfected with CDIP1 siRNAs (si-CDIP1) or negative controls (si-NC). (B) Western blot analysis of ANP and BNP expressions in cardiomyocytes transfected with si-CDIP1-3, si-CDIP1-3 + miR-133b-3p mimic or si-CDIP1-3 + miR-133b-3p inhibitor in the presence of Ang II. (C) Immunofluorescence staining of cTnT (green) in cardiomyocytes transfected with si-CDIP1-3, si-CDIP1-3 + miR-133b-3p mimic or si-CDIP1-3 + miR-133b-3p inhibitor in the presence of Ang II. Nuclei were stained with DAPI (blue). Scale bar: 20 μm. (D) Western blot analysis of Bax, Bcl-2 and cleaved caspase-3 expressions in cardiomyocytes transfected with si-CDIP1-3, si-CDIP1-3 + miR-133b-3p mimic or si-CDIP1-3 + miR-133b-3p inhibitor in the presence of Ang II. The data are presented as the mean ± SD. *P < 0.05, **P < 0.01, ***P < 0.001.

To further evaluate the role of *CDIP1* in cardiomyocyte hypertrophy and apoptosis, we used siRNA to target *CDIP1*. The results of western blot and qRT-PCR analyses revealed that, compared with si- *CDIP1*-1 and si- *CDIP1*-2, si- *CDIP1*-3 efficiently knocked down *CDIP1* expression in cardiomyocytes ( Figure 5A). Thus, si- *CDIP1*-3 was used for all our experiments in this study. We subsequently cotransfected cardiomyocytes treated with Ang II with si- *CDIP1*-3 along with either the miR-133b-3p mimic or inhibitor. The results indicated that transfection with si- *CDIP1*-3 significantly reduced the expression of ANP and BNP. This reduction was further enhanced by cotransfection with the miR-133b-3p mimic. However, cotransfection with the miR-133b-3p inhibitor abrogated this effect ( Figure 5B). A similar trend was also observed in the cardiomyocytes, as indicated by immunofluorescence staining for cTnT ( Figure 5C). Additionally, transfection with si- *CDIP1*-3 abolished the effects of miR-133b-3b on cardiomyocyte apoptosis ( Figure 5D). Taken together, these findings suggest that the overexpression of miR-133b-3p reduces cardiomyocyte hypertrophy and apoptosis through the modulation of *CDIP1*.

## Discussion

Pathologic cardiac hypertrophy can lead to ventricular hypertrophy, dilation, and heart failure, which are among the leading causes of death globally [1]. Ang II has multiple effects on the cardiovascular system, one of the most notable being its role in promoting cardiac hypertrophy [6]. Apoptosis is the main pathological process in cardiac hypertrophy, exerting a significant influence on its occurrence and development [22]. Numerous studies have indicated that Ang II can activate proapoptotic signalling pathways involving increased ROS production, mitochondrial dysfunction, and the activation of MAPKs such as p38 and JNK [23]. These factors can lead to programmed cell death in cardiomyocytes; however, the underlying mechanisms remain unclear. In recent years, accumulating evidence has indicated that miRNAs contribute to the pathological progression of cardiac hypertrophy [24]. This study provides the first data demonstrating that overexpression of miR-133b-3p efficiently alleviates cardiac hypertrophy and apoptosis by targeting *CDIP1*, suggesting a new therapeutic strategy for treating cardiac hypertrophy.

MicroRNAs (miRNAs) are a group of endogenous noncoding single-stranded small-molecule RNAs comprising 21–23 nucleotides [25]. As important regulatory factors in the field of cardiovascular disease, there is increasing evidence that miRNAs are involved in heart development and certain cardiovascular diseases, including cardiac hypertrophy, in both experimental animals and patients [26]. For example, the inhibition of miR-29 could inhibit cardiac hypertrophy *in vitro*, and circulating miR-29 could also serve as a biomarker for predicting cardiac hypertrophy in patients [27]. In addition, miR-1 is abundantly expressed in the heart and plays a protective role against cardiac hypertrophy by targeting several pro-hypertrophic signalling pathways [28]. These results indicate that the deregulation of miRNAs plays critical roles in the progression of cardiac hypertrophy. In recent years, miR-133b has been shown to play tumor-suppressing roles in several malignancies, such as pancreatic cancer, breast cancer, and colorectal cancer [ 29– 31] . Notably, Yu *et al*. [16] compared the expression of seven cardiac- or muscle-specific miRNAs that may be related to heart development in heart tissue from mice and reported that miR-133b-3p was significantly decreased during the postnatal heart growth period. To the best of our knowledge, the role of miR-133b-3p in the pathogenesis of cardiac hypertrophy remains unknown. In this study, we examined the expression and role of miR-133b-3p in an animal model and a cell model of cardiac hypertrophy. MiR-133b-3p expression is markedly decreased in Ang II-infused mice and in cardiomyocytes treated with Ang II. The overexpression of miR-133b-3p can reduce the Ang II-induced increase in cell surface area and the upregulation of hypertrophic markers (ANP and BNP) in cardiomyocytes. It also decreases the expression of the proapoptotic proteins Bax and cleaved caspase-3 while increasing the expression of the antiapoptotic protein Bcl-2. These findings suggested that the overexpression of miR-133b-3p inhibits Ang II-induced cardiomyocyte hypertrophy by suppressing apoptosis. However, a recent study reported that the overexpression of miR-133b-3p promotes the apoptosis of TM3 cells. This apparent contradiction may stem from differences in cellular context and tissue-specific regulatory mechanisms.

Previous studies have largely proposed that miRNAs regulate gene expression at the posttranscriptional level by binding to the 3ʹUTRs of target mRNAs [8]. Recent studies have demonstrated that miR-30d prevents pathological cardiac hypertrophy via negatively regulating its target genes MAP4K4 and GRP78 and inhibiting the prohypertrophic nuclear factor of activated T cells [10]. In addition, miR-142-3p mitigates cardiac hypertrophy by directly inhibiting the expression of the *SH2B1* gene *in vivo* [32]. In this study, we identified a binding site for miR-133b-3p at the 3′UTR of *CDIP1*. We confirmed their interaction by a dual-luciferase reporter assay and demonstrated that the *CDIP1* expression level could be negatively regulated by miR-133b-3p expression. *CDIP1* was identified as a p53 target gene that is upregulated upon DNA damage and is a key downstream effector of p53-dependent apoptosis [33]. It upregulates TNF-α and directs apoptosis upon cell exposure to stress [21]. *CDIP1* has also been reported to be a key signal transducer between mitochondrial apoptosis and ER stress-mediated apoptosis [19]. Moreover, *CDIP1* is abundantly expressed in the heart and may significantly contribute to the development of heart pathologies [34]. Recently, it was reported that silencing the *CDIP1* gene via exosomal miR-21-5p decreases infarct size, improves cardiac function, increases cardiac angiogenesis, and improves myocardial infarction regeneration [35]. However, the involvement of *CDIP1* in cardiac hypertrophy has not yet been explored. Here, we demonstrated for the first time that the expression of *CDIP1* was increased in both an animal model and a cell model of cardiac hypertrophy and that silencing *CDIP1* inhibited cardiac hypertrophy and apoptosis induced by Ang II. Moreover, our rescue experiment suggested that the inhibition of miR-133b-3p reversed the suppressive effects of *CDIP1* silencing on cardiac hypertrophy and apoptosis. These results suggest novel functions of CDIP1 in regulating cardiac hypertrophy.

**Figure 6:**
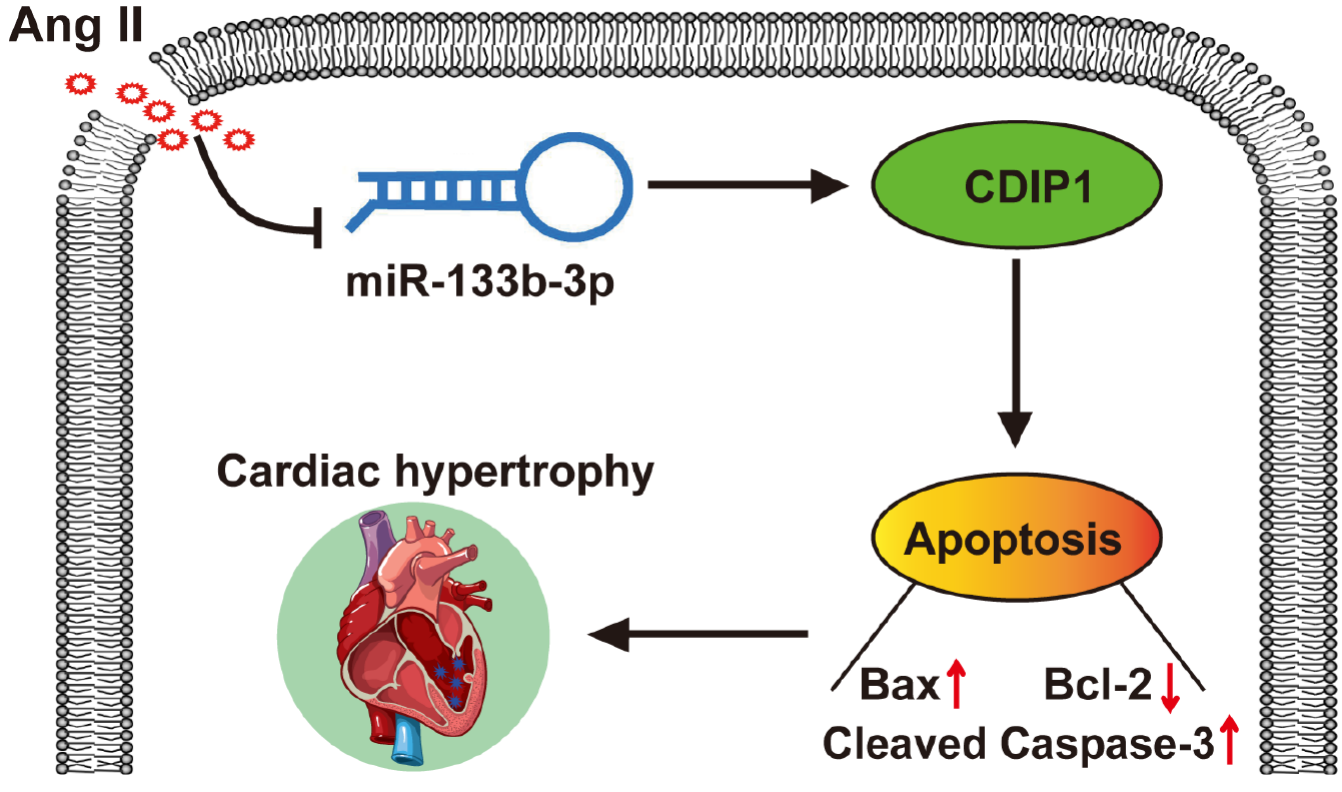
Figure 6 MiR-133b-3p attenuates angiotensin II-induced cardiac hypertrophy through the inhibition of apoptosis by targeting *CDIP1* Downregulation of miR-133b-3p promotes Ang II-induced cardiomyocyte hypertrophy and apoptosis. Mechanistically, miR-133b-3p directly binds to the CDIP1 3′UTR, thereby promoting CDIP1 expression and subsequently enhancing Ang II-induced cardiomyocyte apoptosis.

In summary, we reported that miR-133b-3p is downregulated in Ang II-induced models of cardiac hypertrophy. Overexpression of miR-133b-3p alleviates cardiac hypertrophy and apoptosis induced by Ang II. Additionally, our work corroborates the contribution of the miR-133b-3p/CDIP1 axis to cardiac hypertrophy ( Figure 6). Future investigations are needed to establish a more comprehensive and in-depth understanding of the role played by miR-133b-3p in the hypertrophy process, as well as the detailed mechanisms underlying the regulation of cardiac hypertrophy by miR-133b-3p.
